# Supplementary material for: Cell lineage inference from SNP and scRNA-Seq data
Source: Nucleic Acids Res. 2019 Mar 1;47(10):e56. doi: 10.1093/nar/gkz146 (PMC6547431; doi:10.1093/nar/gkz146)
Supplement: gkz146_Supplemental_File [file gkz146_supplemental_file.pdf]

Supplement-Cell lineage inference from SNP and scRNA-seq

Jun Ding[1], Chieh Lin[2], and Ziv Bar-Joseph *[1,2]

[1]Computational Biology Department, Carnegie Mellon University, PA, United States

[2]Machine Learning Department, Carnegie Mellon University, PA, United States

Contents

Supporting Methods **2**

- 20% cutoff for filtering rare SNPs 2
- Greedy Algorithm to find $P(I)$ 2
- refining SNPs P iteratively 2
- The conditional probability of cell c_i in cluster s based on SNP information 2

Supporting Results **4**

- Comparison with other tools on datasets with the prior knowledge of trajectories . 4
- Predicted SNPs are informative to clustering and trajectory inference 4
- SNP information improves the expression-based scdiff trajectories 5
- SNP information improves the expression-based Monocle trajectories 5
- Predicted SNPs and trajectories of the larger dataset 6
- Predicted SNPs may represent RNA-editing changes 6

Supporting tables **8**

Supporting Figures **9**

*Corresponding author

Supporting Methods

20% cutoff for Rare SNPs

.

To test the robustness of the method in the selection of the cutoff for filtering rare SNPs (potentially enriched with false positives), we have also tried 20% cutoff. As you can see in Supporting Figure S 1 below, the predicted trajectories (tested on the Neuron dataset) are almost identical to the ones of 10% cutoff, Furthermore, 20 out of 28 (71.4%) predicted SNPs under 20% cutoff are also identified under 10% cutoff.

Greedy Algorithm to find $P(I)$

Algorithm 1: Greedy Algorithm to find $P(I)$

Select a SNP $s1$ from X such that $S_{update} = \frac{f(s1,I)}{|I|} - \frac{f(s1,C-I)}{|C-I|}$ is the maximal, add $s1$ to P_I;
do
- $S \leftarrow S_{update}$;
- Select a SNP $s2$ from the remaining SNPs: $X - set(P_I)$ $S_{and} = \frac{f(P_I \wedge s2,I)}{|I|} - \frac{f(P_I \wedge s2,C-I)}{|C-I|}$, $S_{or} = \frac{f(s2 \vee P_I,I)}{|I|} - \frac{f(s2 \vee P_I,C-I)}{|C-I|}$ such that $S_{update} = max(S_{and}, S_{or})$ is the maximal;
- **if** $S_{or} > S_{and}$ **then**
  - $P_I = s2 \vee P_I$;
- **else**
  - $P_I = P_I \wedge s2$;
- **end**

while $S_{update} > S$;
Finally, we will get $P_I = s_1 \vee s_2 \vee s_3, ..., \vee s_m \wedge s_{m+1} \wedge s_{m+2}, ..., s_n$ and then $P(I) = [s_1, s_2, ..., s_n]$

Where X represents all the SNPs, I denotes the cells in the Cluster, C represents all the Cells and $f(P_I, I)$ denotes how many cells in cluster I with SNP set P_I. $P(I)$ is the list of all SNPs in P_I.

refining SNPs P iteratively

Algorithm 2: refining SNPs P iteratively

do
- Cluster the cells using current SNP candidates;
- $P_{update} = \bigcup_{I \in N} P(I)$, where $P(I)$ is identified based on current clustering using Algorithm 1;
- $P_{previous} \leftarrow P_{update}$;
- **do**
  - $P \leftarrow P_{update}$;
  - Choose a SNP s from the remaining SNPs, $s = Argmax_{s \in X-P} Silhouette(P_{update})$, where $P_{update} = P \bigcup s$.
- **while** $Silhouette(P_{update}) > Silhouette(P)$;

while $Silhouette(P) > Silhouette(P_{previous})$;

The conditional probability of cell c_i in cluster s based on SNP information

$$\log ps(c_i|s) = \sum_{x \in P} log(M(c_i, x) * p(x|s) + (1 - M(c_i, x)) * (1 - p(x|s))) \tag{1}$$

where P is the identified SNP list. $M(c_i, x)$ represents whether SNP x is identified in cell c_i as described above. $p(x|s)$ is the probability of snp x identified in cluster s, which is the

percentage of cells with the snp x in the cells of the node. Then we normalized the $ps(ci|s)$ as the following:

$$ps(c_i) = [ps(c_i|s)|s \in Clusters]$$
$$ps_{normalized}(c_i|s) = \frac{ps(c_i|s) - min(ps(c_i))}{max(ps(c_i)) - min(ps(c_i))} \quad (2)$$

Supporting Results

Comparison with existing methods on datasets with the prior knowledge of trajectories

We have compared our methods with Monocle2 on several datasets with some prior knowledge of trajectories. These comparisons demonstrate the advantages of our SNP-based methods. Supporting Figure S3 (A) presents the results of Monocle 2.4.0 on the 2016 neuron reprogramming cells. As can be seen, in these results d2_induced cells are displayed on a separate branch (bottom right) whereas neuron cells are on the top branch. This contradicts the finding of the original study, in which d2_induced cells are shown to be the progenitor of neuron cells. In contrast, SNP-based trajectories correctly order these cells as can be seen in Figure 2. Also, in the results in Supporting Figure S3 d2_induced cells appear after d5_intermediate cells, which is also inconsistent with prior knowledge. Again, SNP results display the correct order for these cells as shown Figure 2. For the liver dataset, we observe that the Monocle and TBSP results agree, even though the SNP results only rely on RNA mutation data. Specifically, as shown in Supporting Figure S3 (B), using Monocle 2 IH and MH cells are clustered together, which is consistent with our SNP-based trajectories (through disagrees with the original study). There are two major branches in this model, the leftmost branch is dominated by LB, MSC and HUVEC cells and the top right branch is dominated by IH and MH cells. This perfectly matches the SNP-based trajectories. For the lung data, we observe a clear trajectory from E14 $\rightarrow$ E16 $\rightarrow$ terminal cells (AT1, AT2, Clara, ciliated) when using Monocle 2. The trajectory improves upon the SNP only trajectories shown in Supporting Figure S4. However, as we show in Supporting Figure S6, when the SNP information is combined with expression information, the combined model which uses the additional SNP data improves upon the expression only model from Monocle 2. For example, in the Monocle 2 results in Supporting Figure S3 (C), Clara and ciliated cells are mixed with other 16, BP and AT1 cells whereas they are clearly separated in the combined model. Also, AT1 and AT2 cells are well-separated in the combined model. We believe that the lower performance accuracy of the SNP only method for the lung data is the result of the very short time series (3 time points, a total of 4 days) which reduces differences between cell types. However, as we just showed the SNP information is still useful and when combined with expression data leads to the most accurate model.

Predicted SNPs are informative to clustering and trajectory inference

Our strategy identified 36, 55, and 33 SNPs for the Neuron data (Treutlein et al., 2016), Liver data (Camp et al., 2017) and Lung data (Treutlein et al., 2014) respectively. Most of the SNPs are associated with specific clusters and the cells in the closer clusters tend to have more similar SNPs. In the Neuron data, almost all SNPs are enriched (found in $> 60\%$ cells of the cluster) in Cluster 1 (dominated by MEFs, the starting cells). On the other hand, only 9 SNPs are enriched (found $> 60\%$ of cells) in Cluster 6 (Neuron cells). SNP 0 -SNP 3 are only enriched in Cluster 6 (Neuron cells) and no other clusters, which could serve as signature SNPs for Cluster 6. While SNP4-SNP 10 are mostly enriched in Cluster 1 (MEFs) and hardly found in all other clusters, which tells that these SNPs are associated with Cluster 1. Similarly, all other clusters can be uniquely marked by a list of SNPs to tell them apart from others. Also, close cell types (clusters) are sharing similar SNP patterns. For example, the SNP distribution for the 4 similar clusters: Cluster 1 (MEF), Cluster 0 (d2_intermediate), Cluster 2 (d2_induced) and Cluster 4(d2_induced) are very similar. In the liver data, such a conclusion also holds. SNP31-38 are only enriched in Cluster 2(dominated by HUVEC cells). SNP49-SNP52 are only enriched in Cluster 0 (MH, IH). SNP 15-20 are only enriched in Cluster 4 (iPSC). Close clusters such as Cluster 4 (iPSC) and Cluster 5 (DE, HE) are having similar SNP patterns. In the Lung data, Cluster 1,4,3 (E14 cells) are having very similar SNP patterns, which are very different to the remaining clusters. As the cell types in the lung data are all relatively close, we can't find many informative SNPs just as indicated in Figure 3, which basically explains why the performance in the lung data is slightly worse. Cluster 5 (Late AT1) and Cluster 0(Early AT1, AT2,BP, Club and ciliated) are having very similar SNP patterns. The only major difference between them are SNP0-SNP2, which are enriched in Cluster 0 but not Cluster 5. SNP0-SNP2 are also enriched in Cluster 1,4,3 (the starting E14 cells), which indicates that Cluster 0 is closer to

the starting cells. This is consistent with the conclusion in (?). Similar results have been found for the mouse blood data (Supporting Figure 10 (B)). In general, the SNP signature for each cluster is quite different and closer clusters tend to have more similar associated SNPs, which indicates that the predicted SNPs are very informative for clustering the cells and further inferring the trajectories.

SNP information improves the expression-based scdiff trajectories

By incorporating the SNP information with the expression, the trajectory inference can be greatly improved.

In the Neuron data, the SNP information re-orders the clusters, making the trajectories more consistent with the prior knowledge in (Treutlein et al., 2016). In the expression only based model, the neuron cluster is a descendant of the cluster with a mixture of Fibroblast, Myocyte and Neuron cells. Also, d5_earlyiN dominant cluster (right bottom) is distant to the neuron dominant cluster. This contradicts the prior knowledge. In the model with both expression and SNP information, the neuron dominant cluster is the descendant to the d2_induced and d5_earlyiN dominant cluster. Also the d5_earlyiN cells are relatively close to the Neuron cells. The SNP+expression based model is more consistent with the trajectory($MEF \rightarrow d2\_intermediate \rightarrow d2\_induced \rightarrow d5\_earlyiN \rightarrow Neuron$) reported in the original study (Treutlein et al., 2016).

In the Liver data, the SNP information also significantly improves the expression only based model(Supporting Figure S 6). Although the cell clustering performance in expression only based model and expression+SNP based model are quite similar. The latter trajectories are more supported by the prior knowledge from the original study (Camp et al., 2017), in which the differentiation should start from iPSC, DE (definitive endoderm) and then diverges to MH(mature hepatocyte-like) (one branch) and LB (liver bud), MSC (mesenchymal stem cells) (another branch). Also, HUVEC (Human umbilical vein endothelial cells) is different from the other liver-specific cells. This is also captured by the SNP integrated model, in which it diverged at the beginning.

In the Lung data, the SNP expression based model is also improved by integrating the SNP information (Supporting Figure S 7). In the expression based model, the AT2 dominant cluster is the child of the AT1/BP cluster while it is a sibling node of the AT1/BP cluster in the SNP+expression model. The latter is more consistent with the trajectories reported in (Treutlein et al., 2014).

SNP information improves the expression-based Monocle trajectories

To determine if the mutation analysis preformed by TBSP can indeed lead to trajectory improvements from other expression-based method, we also used Monocle (version 2.4.0) to reconstruct trajectories for the neuron data we used (for which we have known cell assignments and orders) with and without the use of the mutation data from TBSP. We first applied Monocle to just the expression data (Supporting Figure S 8 left). We next combined the mutations identified by TBSP with the expression data to create a new input for Monocle (For cell cell, we have a vector of binary values to represent whether a certain SNP is present in this cell). To balance the impact of the smaller number of mutations features, we replicated each mutation value 100 times for each cell and concatenated the (replicated) mutation vector with the expression vector to create a new "expression" profile for each cell. We next re-run Monocle on the mutation plus expression input to reconstruct trajectories. The result is presented in Supporting Figure S 8. The major difference between the expression only and expression plus SNP trajectories is the position of d2_induced cells. In the expression + SNP model, d2_induced cells are predicted to be progenitor for descendant cells (e.g. Neuron), consistent with known differentiation order. In contrast, when using only expression data, d2_induced cells are located at a separate branch.

Besides, we used the reconstructed trajectories to compute a pairwise distance between all cells. The distance is calculated as number of different cell types between two cells in the reconstructed trajectories. With this metric, we can calculate a distance matrix for known trajectories (cell orders) M_t, Monocle expression only trajectories M_{m1}, and Monocle expression plus SNP trajectories M_{m2} individually. If we compare M_t with M_{m1}, we see an

average distance difference of 4.158 (smaller the better) and a Pearson Correlation coefficient of 0.668 (larger the better). However, when comparing M_t with M_{m2}, we see an average distance difference of 3.32 and Pearson Correlation of 0.738 (10% improvement). Please refer to Supporting Figure S 9 for the distance matrices.

A Direct comparison of methods using mutations to those relying only on expression data as shown above clearly indicates that the mutations provide additional information for trajectory inference, which is the primary advantage of the method.

Predicted SNPs and trajectories of the larger dataset

To test the scalability of TBSP we have also used it to analyze a larger dataset which profiled close to 3840 Hematopoietic stem/progenitor cells (HSPCs) ($\sim 1.5Mreads/cell$) from mice bone marrow (Nestorowa et al., 2016). While the data was collected over two days in order to study cell differentiation, all HSPCs were pooled before profiling and so unlike the longitudinal datasets discussed above, no time information is available for cells in this dataset. TBSP was still able to identify several significant SNPs for this data and these were used to cluster the cells and derive trajectories. Results are presented in Supporting Figure S 10. Even though it does not use the expression data itself, TBSP derived trajectories agree well with the observation in the original paper of near-continuous differentiation process, from Haematopoietic stem cell/multipotent progenitor to Haematopoietic progenitor cell. Both the differentiation direction and the continuous process are captured by the SNP-based model, in which the trajectory starts from Cluster 0 (60.6% Haematopoietic stem cell/multipotent progenitor cells, 19.1% Long-term Haematopoietic stem cells, 20.3% Haematopoietic progenitor cells), ends at Cluster 1 (86.1% Haematopoietic progenitor cells, 11.2% Haematopoietic stem cell/multipotent progenitor cells, 2.8% Long-term Haematopoietic stem cells) and the percentage of Haematopoietic progenitor cells in the cluster is increasing along the trajectories: 20.3% (cluster 0) $\rightarrow$ 35.7% (cluster 4) $\rightarrow$ 40.3% (cluster 3) $\rightarrow$ 85.8%(cluster 2) $\rightarrow$ 86.1%(cluster 1).

Predicted SNPs may represent RNA-editing changes

The predicted SNPs are found near the Alu elements. For the Neuron data, 17 out of 36 (47.2%) of the predicted SNPs are located within 2000 nucleotides of Alu elements and 29 out of 36 (80.56%) of the predicted SNPs are located within 5000 nucleotides of the Alu elements. We downloaded all 1142278 Alu elements genome wide (2.8G base pairs) from UCSC genome browser (Casper et al., 2017). There are 102 Alu elements found within 5000 bps of the predicted SNPs, which is significantly enriched compared with the background (nearby 100000 bps of each SNP) where we found 1161 Alu elements ($p - value = 1 - pbinom(102 - 1, 36 * 5000, 1161/(100000 * 36)) = 1.16 \times 10^{-7}$). For the Liver data, 37 out of 55 (67.3%) of the predicted SNPs are located within 2000 nucleotides of Alu elements and 49 out of 55 (89.1%) of the predicted SNPs are located within 5000 nucleotides of Alu elements. There are 316 Alu elements found within 5000 bps of the predicted SNPs ($p - value = 1 - pbinom(316 - 1, 55 * 5000, 3595/(100000 * 55) = 0$). For the Lung data, 16 out of 33 (48.5%) of the predicted SNPs are located within 2000 nucleotides of the Alu elements and 26 out of 33 (78.8%) of the predicted SNPs are located within 5000 nucleotides of the Alu elements. There are 82 Alu elements found within 5000 bps of the predicted SNPs ($p - value = 1 - pbinom(82 - 1, 33 * 5000, 862/(100000 * 33) = 8.76 \times 10^{-8}$).

The predicted SNPs are significantly overlapping with the known RNA-editing sites in the RADAR (Ramaswami and Li, 2013) database. Unfortunately, we only have 8823 known RNA-editing sites in mouse, which is extremely limited for further analysis compared to human, in which we have 2576459 known/predicted RNA-editing sites in the RADAR database. Therefore, we examined 55 predicted SNPs in the human Liver data. To calculate the background probability of RADAR RNA-editing sites, we considered the nearby regions (2000bps) of the predicted SNPs instead of genome-wide. There are 237 RADAR RNA editing sites found in the nearby regions of the 55 SNPs: $p_b = 237/(2000 * 55) = 0.00215$. We found that 3 out of 55 SNPs are found in the RADAR database, which is very significant compared to the background with a binomial test p-value: $p - value = 1 - pbinom(3 - 1, 55, 0.00215 = 2.4 \times 10^{-4}$. Besides, we also tried an RNA-editing site prediction tool RED-MEL (Xiong et al., 2017). In the human liver data, we have randomly chosen 30,0000 sites in the genome (the genomic locations with at least 1 aligned reads). For those 300,000 sites, I

found that 376 of them are predicted by RED-ML as the RNA-editing sites with probability >0.5. Therefore, the background probability is : 376/300,000=0.001253. Using the same 0.5 cutoff, 21 out of 55 predicted SNPs are also identified as RNA-editing sites by RED-MEL, which is very significant $p - value = 1 - pbinom(21 - 1, 55, 0.001253) = 0$.

Besides the above analyses, we have also looked at (Macaulay et al., 2015) paper, in which both RNA-seq and DNA-seq data were simultaneously obtained using G&T-seq. We downloaded DNA-seq and RNA-seq for 112 cells from this study and used our method to identify mutations in both. Under the parameters used for other studies we analyzed, we obtained 7 DNA SNPs and 31 SNPs from RNA-seq. **None** of the 31 RNA-seq SNPs were found in the DNA-seq results. Therefore, these mutations are only identified at the RNA but not the DNA level supporting their likely assignments as RNA-editing events. Please see Supporting Table S2 for the list of SNPs identified.

Supporting tables

Supporting Table S 1: SNP-based clustering performance

**Treutlein et al. 2016 Mouse Neuron Data**	MEF	d2_intermediate	d2_induced	d5_intermediate	d5_earlyiN	d5_earlyMyocyte	d5_failedReprog	Fibroblast	Myocyte	Neuron
Cluster 1	61	1	3	0	0	0	0	0	0	0
Cluster 0	3	1	11	0	2	0	0	1	0	1
Cluster 2	12	16	6	0	0	0	0	0	0	1
Cluster 4	0	1	28	1	0	0	0	0	2	0
Cluster 5	4	0	1	9	0	0	12	1	1	1
Cluster 3	3	0	2	12	14	2	1	1	0	2
Cluster 6	0	0	0	1	0	0	0	2	3	27

**Camp et al. 2017 Human Liver data**	iPSC	DE	HE	IH	MH	LB	HUVEC	MSC
Cluster 4	79	3	1	0	0	0	0	0
Cluster 5	0	53	80	18	21	0	0	0
Cluster 0	0	10	3	50	48	16	0	0
Cluster 3	0	1	27	12	12	36	0	1
Cluster 2	0	0	0	0	0	52	74	0
Cluster 1	0	0	0	0	0	65	0	103

**Treutlein et al. 2014 Mouse Lung data**	E14	AT1	Club	ciliated	BP	AT2	E16
Cluster 1	26	0	0	0	0	0	0
Cluster 4	7	0	0	0	0	0	0
Cluster 3	11	0	0	0	0	0	0
Cluster 0	0	12	9	3	6	8	0
Cluster 2	0	4	1	0	0	1	24
Cluster 5	0	25	1	9	7	3	0

The table provides information on the cells assigned to each cluster based on SNP information only. As can be seen, 61 out of 65 (93.8%) cells in Cluster 1 are MEF cells and 28 out of 32(87.5%) cells in Cluster 4 are d2_induced cells. Generally speaking the clustering based on SNP information is consistent with known cell types.

Supporting Table S 2: List of identified SNPs from G&T-seq data

List of SNPs found in DNA-seq data	List of SNPs found in RNA-seq data
chr6,103649210	chr17,17378510
chr14,19418819	chrX,152910073
chr2,98665093	chr7,45827612
chr14,19417330	chr17,39844879
chr2,98666307	chr17,39848255
chr2,98666664	chr9,37649418
chr2,98662904	chr9,64304427
	chr7,97718637
	chr11,94756642
	chr3,95947248
	chr7,118110013
	chr7,118127190
	chr11,60641946
	chr8,84868043
	chr8,84974212
	chr8,70510145
	chr8,70531055
	chr8,70133263
	chr14,8026348
	chr14,18269146
	chr4,147258755
	chr7,15834715
	chr7,99481761
	chr7,3706741
	chr7,99481717
	chr9,44408787
	chr7,99479976
	chr7,3706702
	chr7,99479928
	chr9,44408721
	chr7,66089057

Supporting Figures

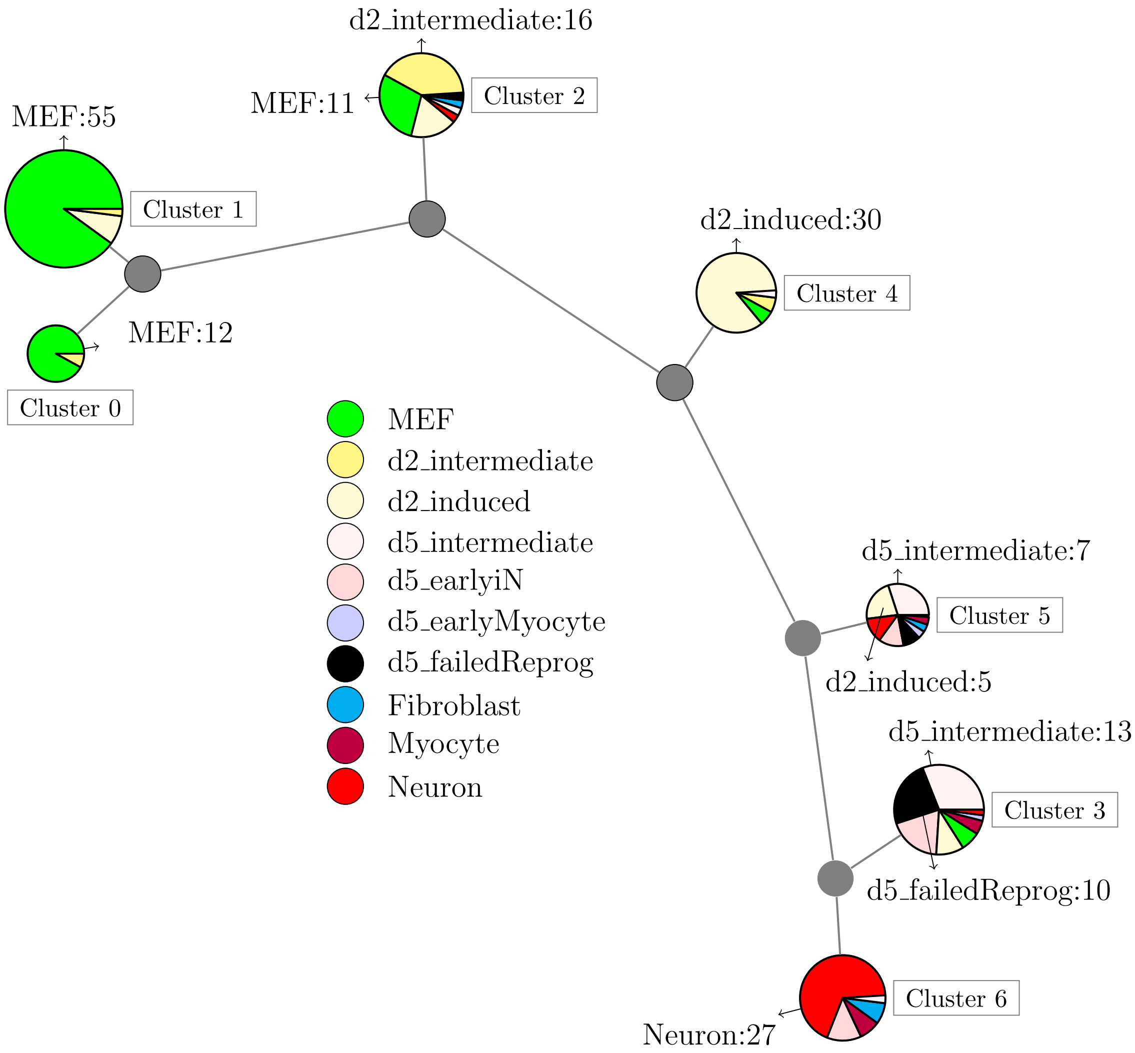

Supporting Figure S 1: **predicted trajectories under 20% cutoff for filtering rare SNPs.** The predicted trajectories are very similar to those under 10% cutoff as shown in Figure 2 in the main text.

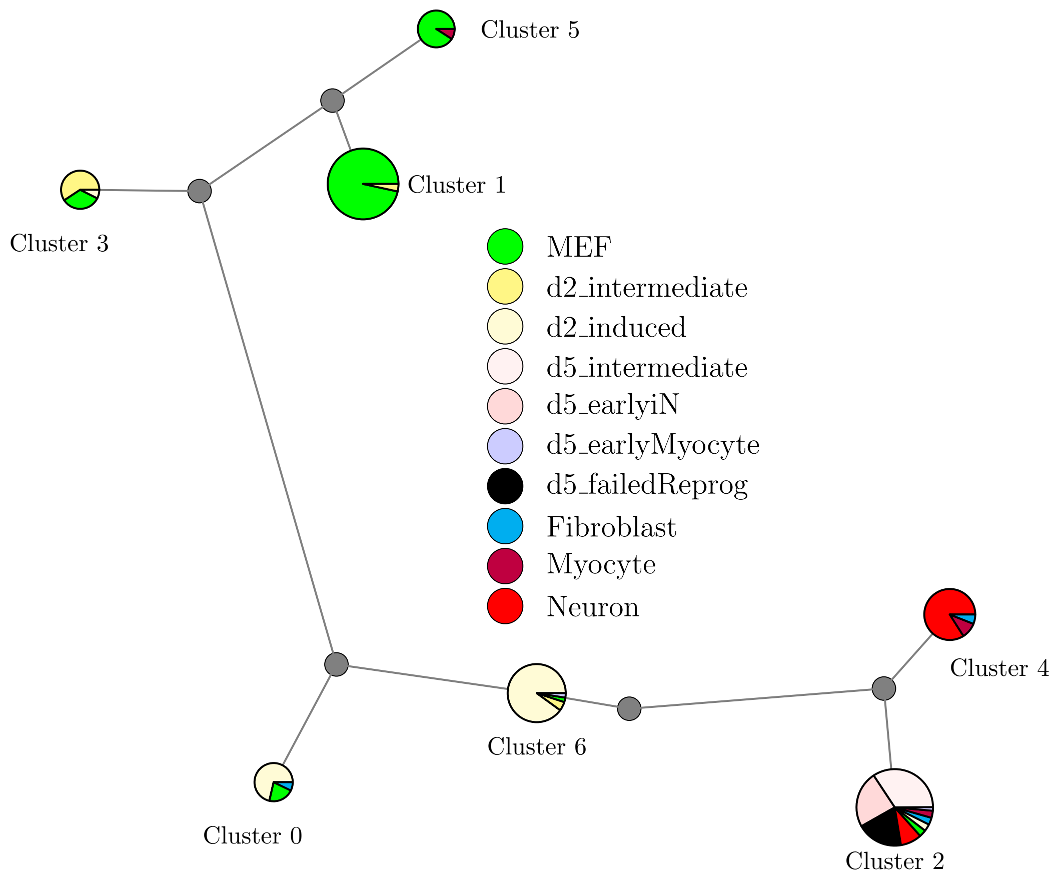

Supporting Figure S 2: **Trajectories inferred using all SNPs including potentially expression-redundant ones.** Many SNPs whose residing genes are only expressing in a subset of cells and thus those SNPs could be potentially redundant to gene expression. The cell trajectories can be slightly improved if including those potentially redundant SNPs. Results, presented above, indicate that the updated trajectories are slightly better when using all SNPs. First, the closest cluster to MEF cells is the d2_intermedidate dominated cluster in this model while it is d2_induced in the original trajectories. Second, the cells are clustered better. d5_intermediate cells are mostly enriched in node 2 in the above model while they are widely distributed across multiple clusters in the original trajectories. d2_indcued cells are also better clustered. In TBSP, we provided options for users to include/exclude potentially 'expression-redundant' SNPs using -b parameter.

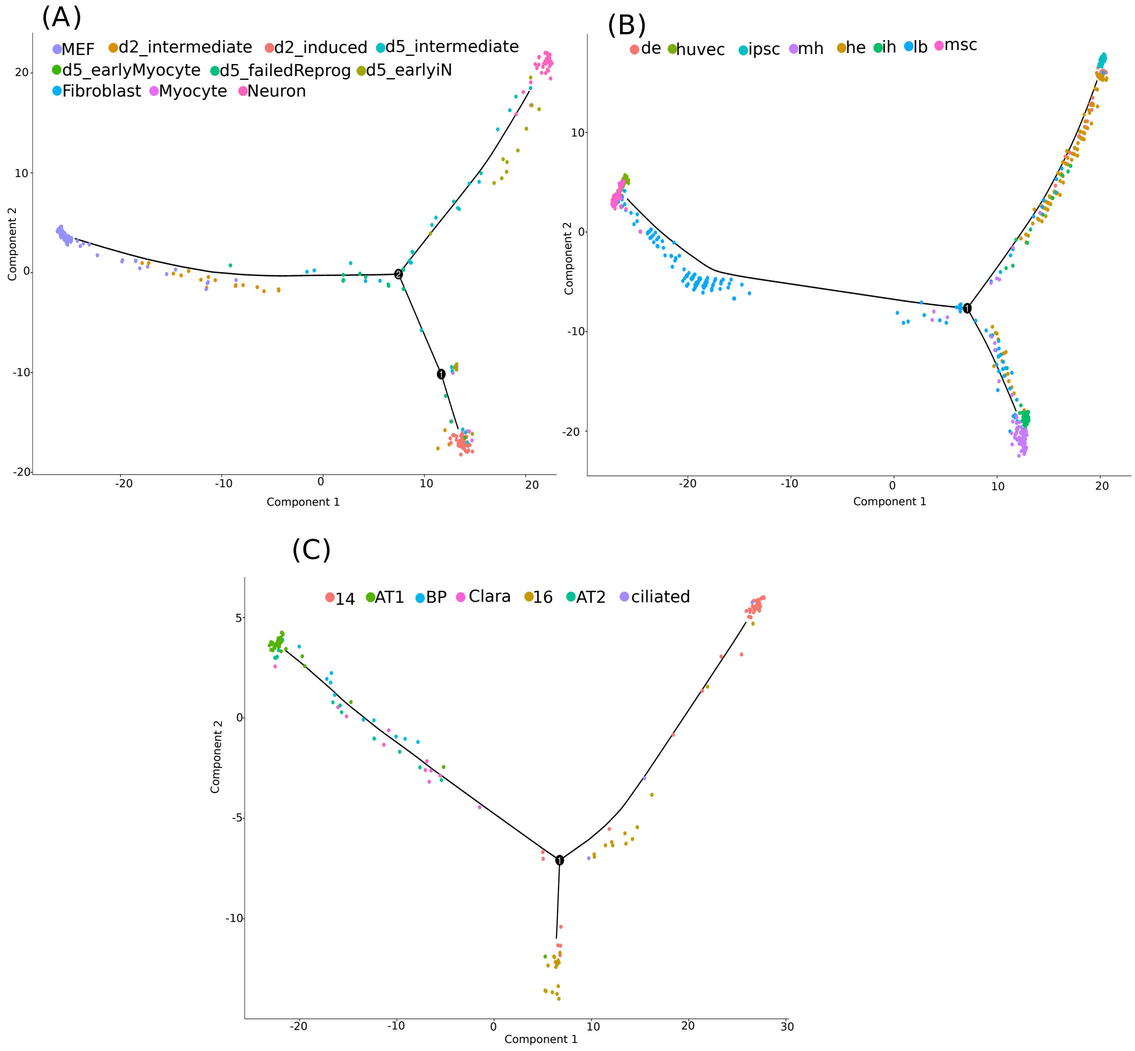

Supporting Figure S 3: **Comparison with monocle 2.** (A) Monocle results on the neuron reprogramming data. As can be seen, d2_induced cells are displayed on a separate branch (bottom right) whereas neuron cells are on the top branch, This contradicts the finding of the original study, in which d2_induced cells are reported to be the progenitor of neuron cells. Also, d2_induced cells appear after d5_intermediate cells, which is also inconsistent with prior knowledge. In contrast, SNP-based trajectories correctly order these cells as shown in Figure 2. (B) Monocle results on the Liver data. We observe that Monocle and SNP results agree well. IH and MH cells are clustered together using Monocle 2, which is consistent with our SNP-based trajectories as can be seen in Figure 2 (through disagrees with the original study). There are two major branches in this model, the leftmost branch is dominated by LB, MSC and HUVEC cells and the top right branch is dominated by IH and MH cells. This perfectly matches the SNP-based trajectories. (C) Monocle results on the lung data. From the lung monocle results, we observe a clear trajectory from E14 $\rightarrow$ E16 $\rightarrow$ terminal cells. This trajectory improves upon the SNP only trajectories as shown in Supporting Figure S 4. However, as we shown in Supporting Figure S 7, when the SNP information is combined with the expression information, the combined model improves upon the expression only model from Monocle 2. For example, Clara and ciliated cells are mixed with other 16, BP or AT1 cells whereas in the combined model, they are clearly separated. Also, AT1 and AT2 cells are well-separated in the combined model.

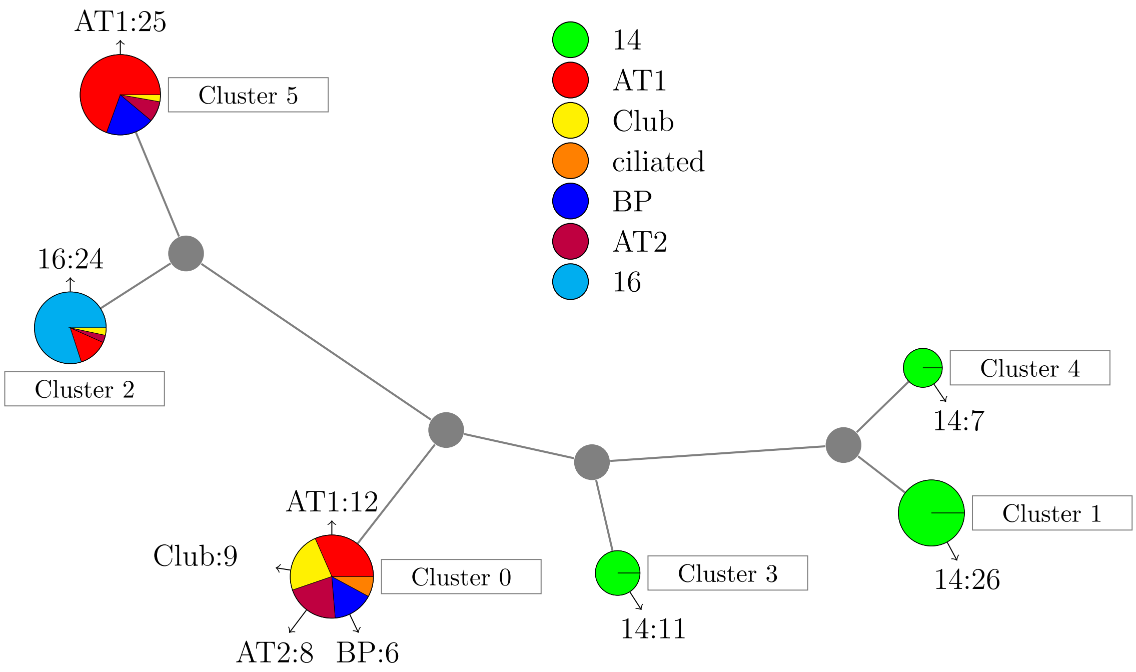

Supporting Figure S 4: **predicted trajectories of the 2014 Treutlein et.al. lung data based on SNPs.** The first time point (E14.5) is associated with a number of unique clusters (1, 3 and 4) residing in the beginning of the tree while more mature epithelial cells (mainly Bi-potential Progenitors (BP), Alveolar Type 2 and Ciliated cells) are clustered together afterwards and the last to branch are Type 1 cells. However, in this model the method incorrectly assigns the E16.5 time point to a later branching location than its actual position in the process.

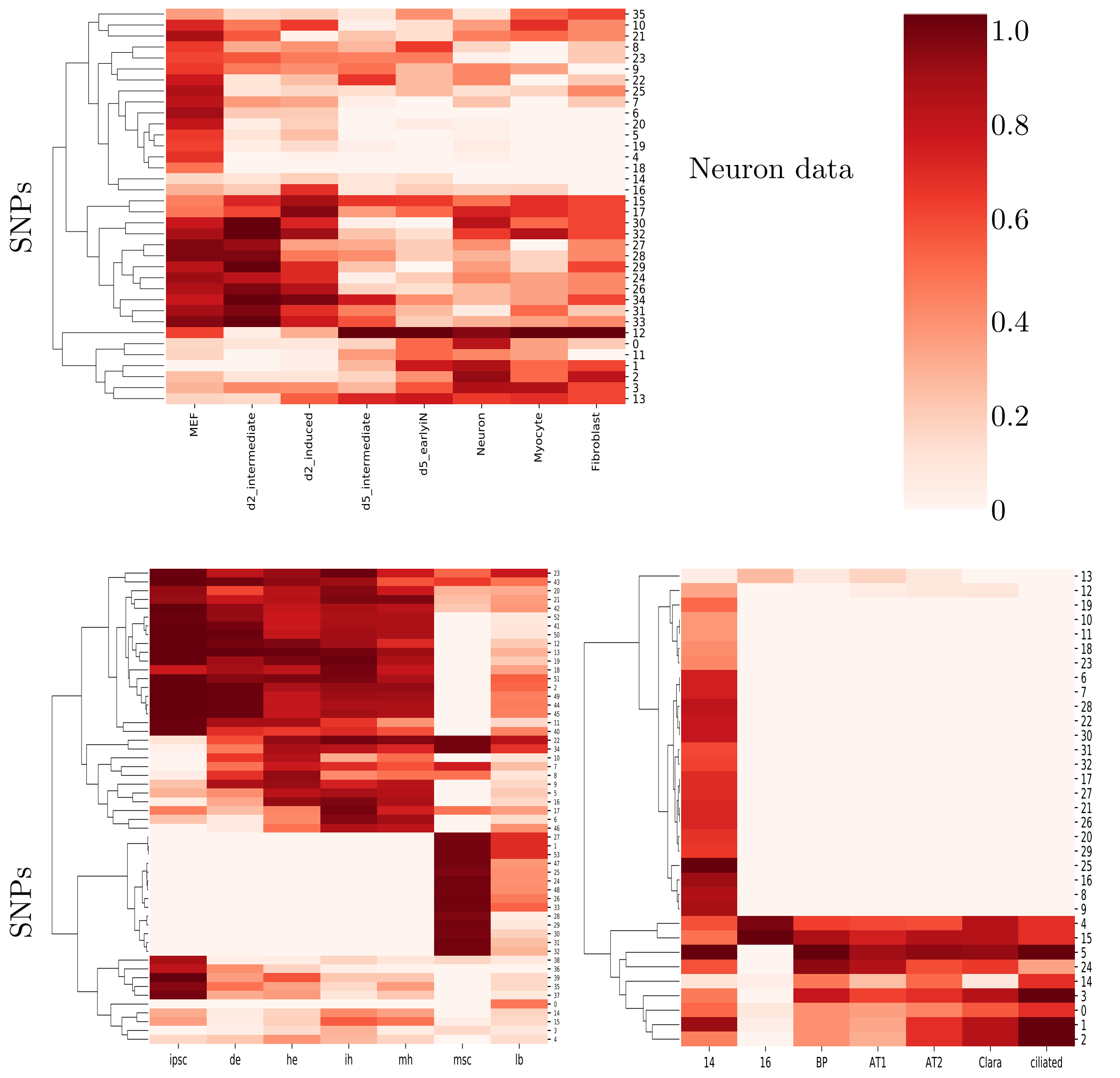

Supporting Figure S 5: **SNP distribution along the known trajectories.** For the neuron data we see several mutations that are only associated with the initial state, We also see some cell types (e.g., d2_intermediate and d2_induced or d5_earlyiN and Neuron) that share the same mutations while other types (for example, Fibroblasts) do not. This supports their use in unsupervised model reconstruction. For the liver data we observe the main difference between MSC, LB cells and IH, MH cells. iPSC cells, as the starting cells, are also displaying a subset of unique SNPs. For the lung data, we observe a clear separation between progenitor cells E14 and terminal cells (AT1, AT2, Clara, and ciliated).

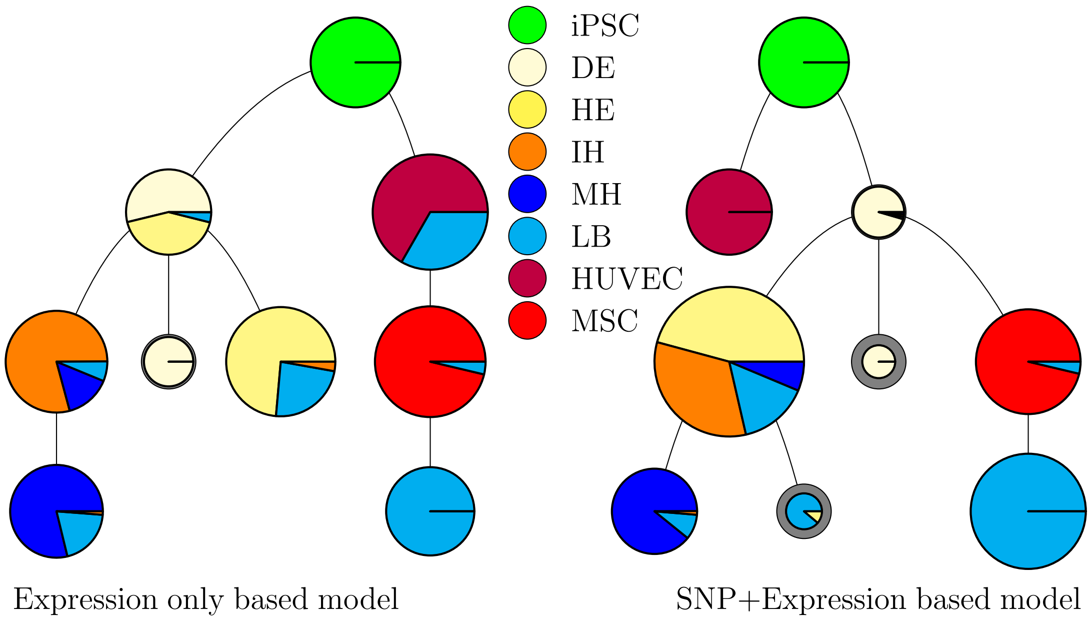

Supporting Figure S 6: **The expression and SNP based trajectory inference for the human Liver data.** The SNP+Expression based trajectories are more supported by the prior knowledge from the original study (Camp et al., 2017), in which the differentiation should start from iPSC, DE (definitive endoderm) and then diverges to MH(mature hepatocyte-like) (one branch) and LB (liver bud), MSC (mesenchymal stem cells) (another branch). Also, HUVEC (Human umbilical vein endothelial cells) is different from the other liver-specific cells. This is also captured by the SNP integrated model, in which it diverged at the beginning.

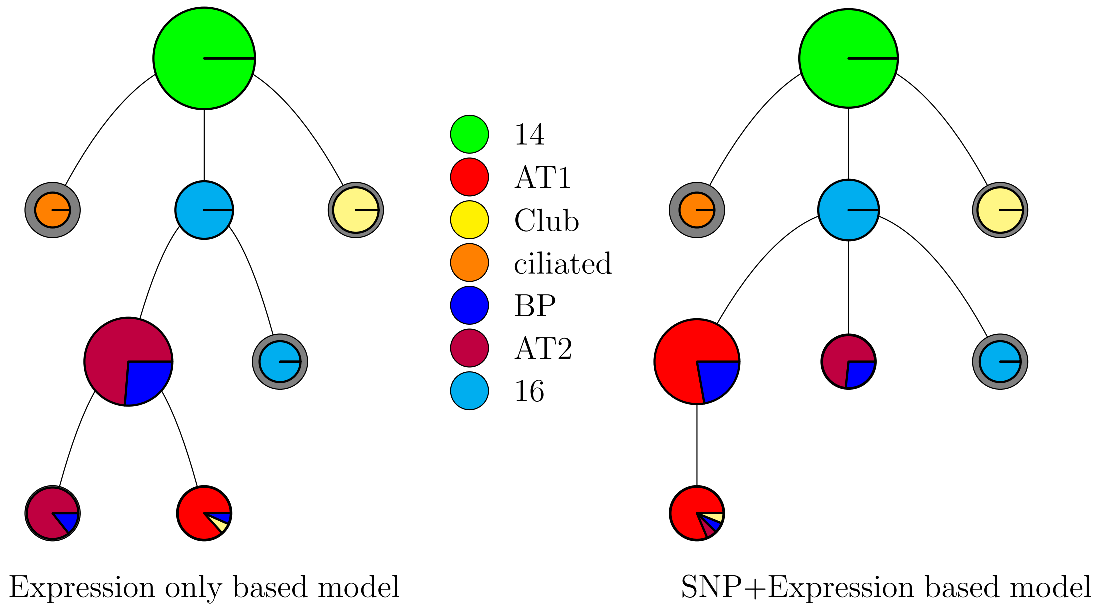

Supporting Figure S 7: **The expression and SNP based trajectory inference for the Mouse Lung data.** The expression based model is also improved by integrating the SNP information. In the expression based model, the AT2 dominant cluster is the child of the AT1/BP cluster while it is sibling node of the AT1/BP cluster in the SNP+expression model. The latter is more consistent with the trajectories reported in (Treutlein et al., 2014).

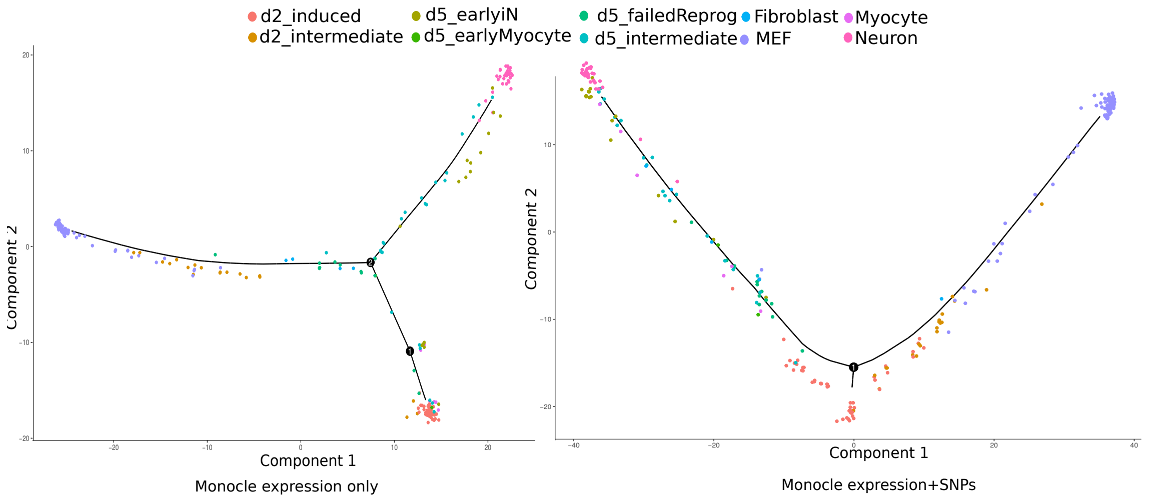

Supporting Figure S 8: **Monocle trajectories with/without using SNPs from TBSP.** Left: Monocle trajectories using only the expression data. Right: Monocle trajectories using the expression data plus SNPs from TBSP.

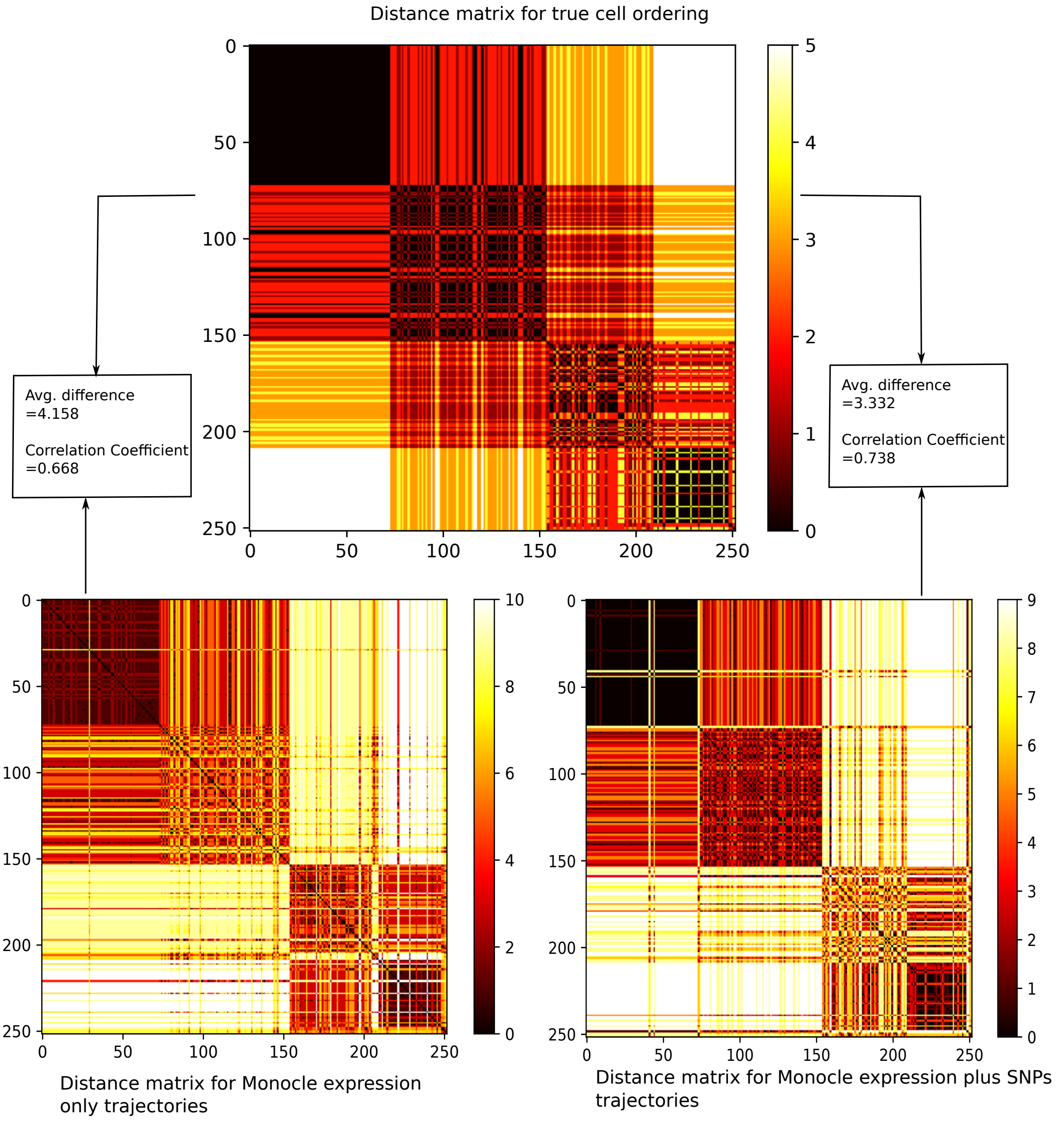

Supporting Figure S 9: **The pair-wise distance matrix between cells.** The pair-wise distance is measured as number of different cell types between cells in given trajectories. Top (M_t): the distance matrix calculated based on the true cell ordering from prior knowledge. Bottom left(M_{m1}): the distance matrix calculated based on Monocle expression only trajectories. Bottom right (M_{m2}): the distance matrix calculated based on Monocle (expression + TBSP SNPs) trajectories. The average distance difference between M_t and M_{m1} is 4.158 and the Pearson correlation coefficient is 0.668. The average distance difference between M_t and M_{m2} is 3.332 and the Pearson correlation coefficient is 0.738.

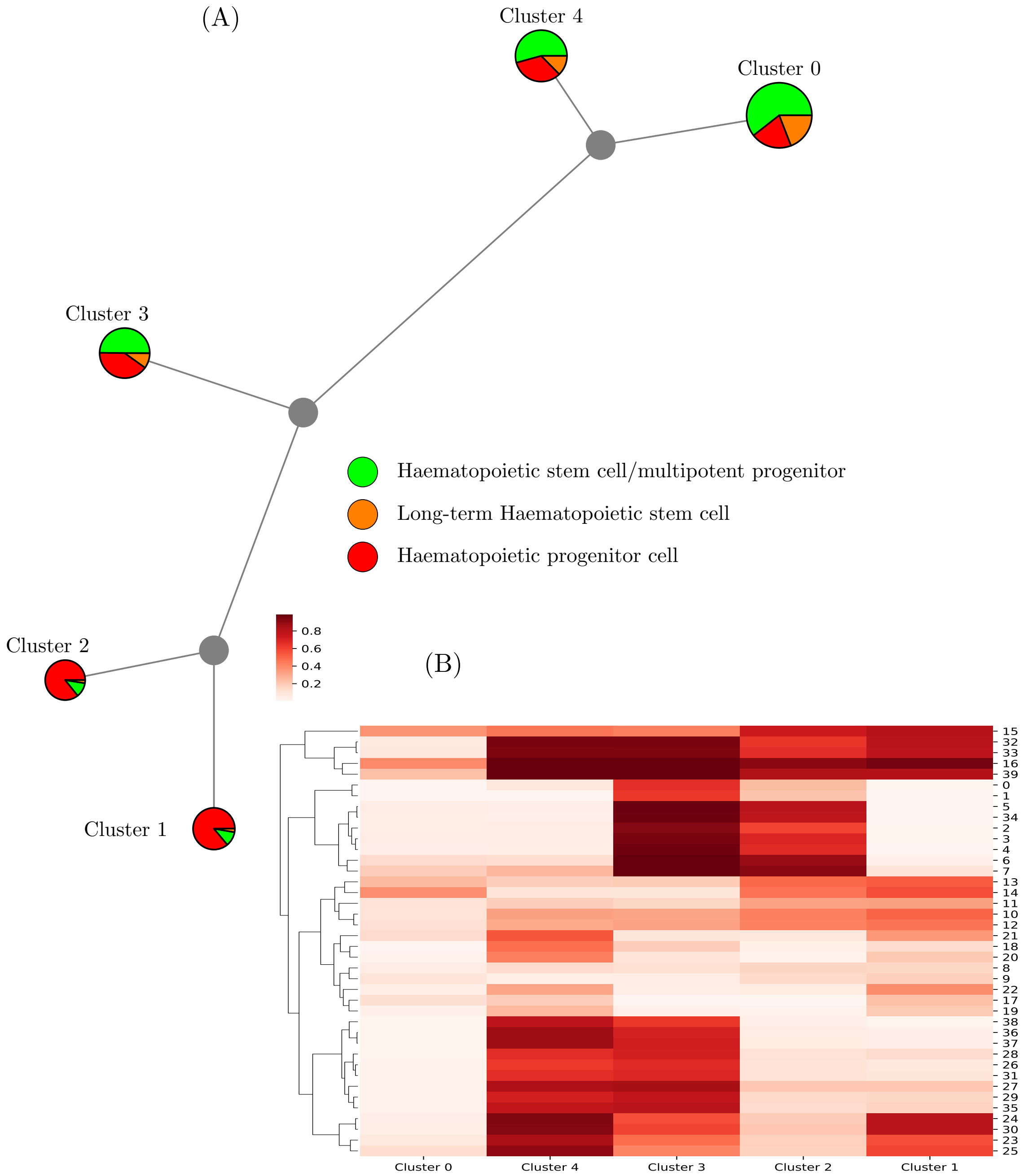

Supporting Figure S 10: **The SNP-based results for the mouse blood data.** (A) The SNP-based trajectory of the mouse blood data. Multipotent progenitor percentage in the cluster is decreasing along the trajectory while the percentage of Haematopoietic stem cell is increasing. (B) The distribution of predicted SNPs in the cluster. For each SNP, the plot shows how many percentages of cells in each cluster with that SNP. Each Cluster has its unique SNP markers and adjacent clusters alone the trajectory share relatively similar SNP markers.

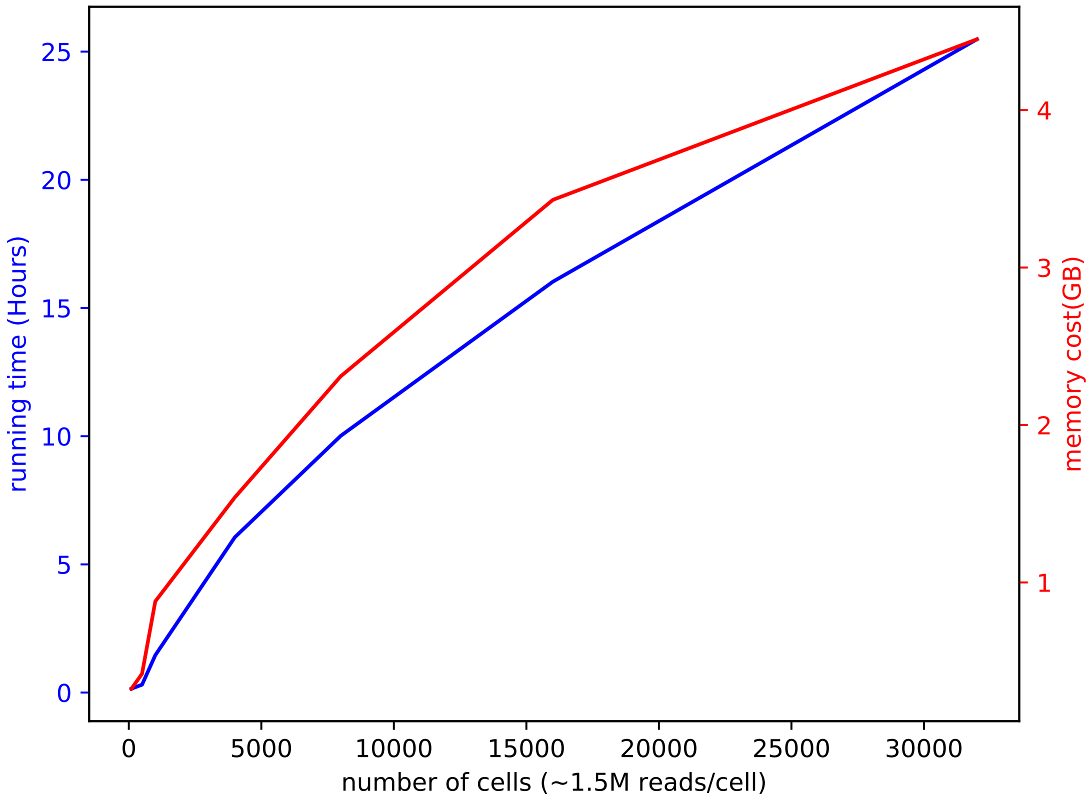

Supporting Figure S 11: **TBSP running time and memory cost.** We simulated up to 30000 cells with 1.5M reads on average for each cell. For such dataset with 30000 cells ($\sim$ 1.5M reads/cell), TBSP takes less than 24.48 hours on an @Interl(R) Xeon(R) CPU E3-1225 v5 @3.3GHz and 4.45G RAM. Note, the preprocessing time (Reads mapping and SNP calling) was not considered here.

References

Camp, J. G., Sekine, K., Gerber, T., Loeffler-Wirth, H., Binder, H., Gac, M., Kanton, S., Kageyama, J., Damm, G., Seehofer, D., et al. (2017). Multilineage communication regulates human liver bud development from pluripotency. *Nature*, 546(7659):533.

Casper, J., Zweig, A. S., Villarreal, C., Tyner, C., Speir, M. L., Rosenbloom, K. R., Raney, B. J., Lee, C. M., Lee, B. T., Karolchik, D., et al. (2017). The ucsc genome browser database: 2018 update. *Nucleic acids research*, 46(D1):D762–D769.

Macaulay, I. C., Haerty, W., Kumar, P., Li, Y. I., Hu, T. X., Teng, M. J., Goolam, M., Saurat, N., Coupland, P., Shirley, L. M., et al. (2015). G&t-seq: parallel sequencing of single-cell genomes and transcriptomes. *Nature methods*, 12(6):519.

Nestorowa, S., Hamey, F. K., Sala, B. P., Diamanti, E., Shepherd, M., Laurenti, E., Wilson, N. K., Kent, D. G., and Göttgens, B. (2016). A single cell resolution map of mouse haematopoietic stem and progenitor cell differentiation. *Blood*, pages blood–2016.

Ramaswami, G. and Li, J. B. (2013). Radar: a rigorously annotated database of a-to-i rna editing. *Nucleic acids research*, 42(D1):D109–D113.

Treutlein, B., Brownfield, D. G., Wu, A. R., Neff, N. F., Mantalas, G. L., Espinoza, F. H., Desai, T. J., Krasnow, M. A., and Quake, S. R. (2014). Reconstructing lineage hierarchies of the distal lung epithelium using single-cell rna-seq. *Nature*, 509(7500):371.

Treutlein, B., Lee, Q. Y., Camp, J. G., Mall, M., Koh, W., Shariati, S. A. M., Sim, S., Neff, N. F., Skotheim, J. M., Wernig, M., et al. (2016). Dissecting direct reprogramming from fibroblast to neuron using single-cell rna-seq. *Nature*, 534(7607):391.

Xiong, H., Liu, D., Li, Q., Lei, M., Xu, L., Wu, L., Wang, Z., Ren, S., Li, W., Xia, M., et al. (2017). Red-ml: a novel, effective rna editing detection method based on machine learning. *Gigascience*, 6(5):1–8.
